# Evidence of Maternal–Fetal Transmission of *Parachlamydia acanthamoebae*

**DOI:** 10.3201/eid1501.080911

**Published:** 2009-01

**Authors:** David Baud, Genevieve Goy, Stefan Gerber, Yvan Vial, Patrick Hohlfeld, Gilbert Greub

**Affiliations:** Institute of Microbiology, Lausanne, Switzerland (D. Baud, G. Goy, G. Greub); University Hospital of Lausanne, Lausanne, Switzerland (D. Baud, S. Gerber, Y. Vial, P. Hohlfeld)

**Keywords:** parachlamydia, Chlamydia-like organism, intrauterine infection, adverse pregnancy outcome, Chlamydia, letter

**To the Editor:** *Parachlamydia acanthamoebae* is a recently identified agent of pneumonia (*1*–*3*) and has been linked to adverse pregnancy outcomes, including human miscarriage and bovine abortion (*4*,*5*). Parachlamydial sequences have also been detected in human cervical smears (*4*) and in guinea pig inclusion conjunctivitis (*6*). We present direct evidence of maternal–fetal transmission of *P. acanthamoebae*.

We tested 78 amniotic fluid samples from patients who delivered prematurely (defined as spontaneous delivery before 37 weeks of gestation) at the Centre Hospitalier Universitaire Vaudois, Lausanne, Switzerland, from 2003 to 2006. DNA was extracted by using the QIAampDNA Mini kit (QIAGEN, Hilden, Germany) and was tested by using a specific *Parachlamydia* real-time PCR (*7*). One positive sample (threshold cycle of 34.2) was confirmed by using the 16SigF-Rp2Chlam PCR, which targets a large DNA segment of the 16S rRNA gene (*8*). Because these 2 PCRs target different DNA segments, the positive PCR results were not due to PCR contamination with amplicons. The sequence exhibited 99.6% similarity with *P. acanthamoebae* strain Hall’s coccus (*1*) (GenBank accession no. AF366365).

The sample was obtained from a 29-year-old woman during her second pregnancy, at 16 weeks of gestation. Amniocentesis was performed because a first-trimester test suggested a Down syndrome risk of 1/100. At the time of amniocentesis, the patient had cough and flu-like symptoms of 3 weeks’ duration, which resolved spontaneously in a few weeks. Cytogenetic analysis showed a normal 46XX karyotype; amniotic fluid culture remained sterile. The pregnancy ended prematurely at 35 weeks and 6 days with the vaginal delivery of a 2,060-g newborn (<5th percentile). The mother and child had an uneventful hospital course.

The role of *Parachlamydia* as the etiologic agent of premature labor and intrauterine growth retardation is likely because 1) all vaginal, placental, and urinary cultures were negative; 2) results of routine serologic tests were negative; and 3) only *Parachlamydia* was detected in the amniotic fluid. Intrauterine infection caused by *Parachlamydia* spp. may be chronic and asymptomatic until adverse pregnancy outcomes occur (*4*).

The infection of this pregnant woman might have occurred through zoonotic contact through her work as a butcher in a rural area known for cattle breeding. Of interest, a recent study of 482 healthy Swiss men found 3 who were seropositive for *Parachlamydia* sp.*,* and all 3 came from the same rural area near Lausanne (within <20-km radius) (*9*). Moreover, the patient owns 2 guinea pigs, potential vectors of the bacterium (*6*). Other modes of transmission are possible, e.g., contaminated water (free-living amebae may serve as hosts for *Parachlamydia* spp. and are widespread in water networks) and ingestion of undercooked meat or contaminated cow milk.

A plausible pathogenic scenario for this case of possible maternal–fetal transmission of *P. acanthamoebae* might include bacteremia in the context of a lung infection. This could have resulted in intrauterine infection and intrauterine growth restriction.
